# Food protein-induced enterocolitis syndrome: Healthcare utilization and referral patterns among a pediatric cohort

**DOI:** 10.3389/falgy.2023.1102410

**Published:** 2023-02-10

**Authors:** Jennifer Pier, Theresa Bingemann, Jasdeep Badwal, Daniel Rosloff, Muhammad Pasha, Hongyue Wang, Jeanne M. Lomas, Kirsi M. Järvinen

**Affiliations:** ^1^ Division of Allergy and Immunology, University of Rochester Medical Center, Rochester, NY, United States; ^2^ Division of Allergy and Immunology, Albany Medical Center, Albany, NY, United States; ^3^ Department of Biostatics and Computational Biology, University of Rochester Medical Center, Rochester, NY, United States

**Keywords:** food allergy, FPIES, non-IgE mediated food allergy, vomiting, healthcare utilization

## Abstract

**Background:**

Food protein–induced enterocolitis syndrome (FPIES) is a non-IgE mediated food allergy characterized by delayed, repetitive vomiting. FPIES is improving in recognition; however, there remains a lag in diagnosis. This study aimed to further explore this lag, as well as referral patterns and healthcare utilization, to help determine areas for earlier recognition.

**Methods:**

A retrospective chart review of pediatric FPIES patients at two hospital systems in New York was completed. Charts were reviewed for FPIES episodes and healthcare visits prior to diagnosis, and reason/source of referral to an allergist. A cohort of patients with IgE-mediated food allergy was reviewed for comparison of demographics and the time to the diagnosis.

**Results:**

In total, 110 patients with FPIES were identified. The median time to diagnosis was 3 months, vs. 2 months in IgE-mediated food allergy (*p* < 0.05). Most referrals were from the pediatrician (68%) or gastroenterology (28%), none were from the ED. The most common reason for referral was concern of IgE-mediated allergy (51%), followed by FPIES (35%). There was a statistically significant difference in race/ethnicity between the FPIES cohort and IgE-mediated food allergy group (*p* < 0.0001), with a greater proportion of Caucasian patients in FPIES vs. IgE-mediated food allergy cohort.

**Conclusion:**

This study demonstrates a lag in the diagnosis of FPIES and a lack of recognition outside of the allergy community, as only one-third of patients were considered to have FPIES prior to an allergy evaluation.

## Introduction

Food protein-induced enterocolitis syndrome (FPIES) is a non-IgE mediated food allergy. Acute form of FPIES reactions are characterized by episodes of repetitive vomiting and associated lethargy, occurring about one to four hours after ingestion of the trigger food, with the possible development of hypovolemic shock due to dehydration (1). The chronic form of FPIES occurs with continual ingestion of the trigger food and most commonly manifests as chronic diarrhea, emesis, and poor growth. Most FPIES occurs in very early childhood, but the condition can also develop in adults. The most commonly implicated foods in children include cow's milk, soy, oat and rice, although there are geographical variations (1). In adults, seafood is the most common trigger. FPIES is a clinical diagnosis. In 2017, consensus guidelines were published, establishing major and minor criteria for the diagnosis of both acute and chronic FPIES (2). Treatment remains avoidance of the trigger food. Antiemetics and intravenous hydration can be used for moderate or severe acute reactions. Fortunately, prognosis is very good, with most childhood FPIES cases resolving by school age, though there are reports of symptoms continuing into adulthood.

The recognition of FPIES has greatly increased over the last few years; however, the epidemiology and pathophysiology remain poorly understood. Birth cohort studies from Israel and Spain estimated the prevalence to be 0.34% for cow's milk FPIES and 0.7% for all FPIES, respectively (3, 4). A recent survey in the United States estimated the prevalence to be about 0.51% and may affect as many as 900,000 individuals in the United States, including 0.5% of children (5). These studies show that FPIES may be more prevalent than previously thought. Additionally, despite improved recognition and awareness over the last few years, there still remains a delay in diagnosis. Blackman et al. performed a retrospective chart review of pediatric FPIES patients at Texas Children's Hospital and found a 6-month delay in diagnosis (6). Ludman et al. retrospectively observed that FPIES patients who presented to a tertiary pediatric allergy clinic in London experienced a 12-month delay in diagnosis (7). A recent cohort study from Australia over the last decade also found a 4-month delay among their patients (8).

In addition to a prolonged length to diagnosis, patients may also experience a number of FPIES episodes prior to diagnosis. Blackman et al. and Mehr et al. found that 32% of patients and 20% of patient, respectively, had up to four FPIES episodes prior to diagnosis (6, 9). Ludman et al. found that in the United Kingdom cohort most were seen initially by their primary physician for their symptoms and were seen an average of two times prior to referral to an allergist (7). These studies show that FPIES remains underrecognized and that earlier recognition is likely to be accomplished with the general medical community.

To our knowledge, there are currently no published studies that evaluate referral patterns related to FPIES within the United States. Additionally, there has not previously been a comparison between length to diagnosis of FPIES vs. IgE-mediated food allergy. This study aimed to provide insight into the path that patients take to diagnosis of FPIES, including length to diagnosis, healthcare visits prior to diagnosis, and specialty referrals.

## Materials and methods

A retrospective chart review was performed for pediatric patients (< 18 years of age) with the diagnosis of FPIES at two hospital systems in Upstate New York (University of Rochester Medical Center (UR), and Rochester Regional Health (RRH) from January 2016 to June 2019. Patients received a clinical diagnosis of FPIES through an allergist based on the currently accepted major and minor criteria. The research protocol was approved by the Research Subject Review Board at each institution. Medical records were reviewed for gender, race, birth history, feeding history, family history of atopic disorders, personal history of atopic disorders, age of symptom onset, age of diagnosis, trigger foods, resolution of symptoms, prior evaluation/treatment or emergency department visits, and referring provider/diagnosis. A control cohort of pediatric patients with IgE-mediated food allergy seen at UR during the same time frame, chosen at random, was used for comparison of demographic data, age of symptom onset, and age of diagnosis.

The analyses were performed using version 9.4 of the SAS System for Windows (SAS institute Inc., Cary, NC, United States). Two sample t test or Wilcoxon Rank Sum test was used to compare continuous variables between patients with FPIES and those with IgE-mediated food allergy, where appropriate. Chi-square or Fisher's exact test was used to compare categorical variables. All tests were two sided and a *p* < 0.05 was considered statistically significant.

## Results

### Differences in cohort characteristics between FPIES and IgE-mediated food allergy

A total of 110 patients with FPIES were identified among the two sites during the time frame. Of the patients identified, 96% had acute FPIES. All patients had a history of vomiting episodes. In addition, 51 patients (46%) reported lethargy, 35 patients (29%) reported diarrhea, and 16 patients (15%) reported skin color changes. Other symptoms noted during the episodes included bloody stools, hypotonia, rash, and abdominal distention.

The demographics for the complete cohort and per each site are shown in Table 1. There were no statistically significant differences between the two sites. The control group includes 100 subjects with IgE-mediated food allergy and is also shown in this Table. There were no statistically significant differences between the cohort and control group in regard to gender, term of birth, mode of birth, birth complications, or feeding. There was a statistically significant difference in race/ethnicity between the FPIES cohort and IgE-mediated food allergy group (*p* < 0.0001), with a greater proportion of Caucasian patients in FPIES vs. IgE-mediated food allergy cohort.

**Table 1:** Demographics and characteristics of the FPIES cohorts at university of Rochester medical center (UR) and Rochester regional health (RRH) and a control group (UR IgE-mediated food allergy).

	UR (47)	RRH (63)	Total (110)	IgE-FA (100)	*p*-value^+^
Male	23 (49%)	31 (49%)	54	53	0.53
Female	24 (51%)	32 (51%)	56	47	
Race/Ethnicity					<0.0001
White	44 (94%)	48 (76%)	92	68	
Black	1 (2%)	0	1	17	
Hispanic	1 (2%)	4 (6%)	5	4	
Asian	0	1 (2%)	1	4	
Birth History
Term	44 (94%)	55 (87%)	99	88	0.66
C-Section	15 (68%)	30 (52%)	45	33	0.27
Complications^a^	10 (21%)	7 (11%)	17	13	0.59
Formula (any)	26 (55%)	22 (35%)	48	49	0.39
Time to diagnosis in months (median)	4	3		2	<0.05

^+^ Comparison done between FPIES cohorts (Total) and IgE-mediated food allergy.

^a^ Complications include intrauterine growth restriction, gestational hypertension, necrotizing enterocolitis, group B streptococcal infection, sepsis, prematurity, meconium aspiration, and pneumonia.

The average age of symptom onset was 9 months. The median length to diagnosis of FPIES was 3 months compared to 2 months for IgE-mediated allergy (*p* < 0.05, Table 1). There was no difference in time to diagnosis between those with liquid FPIES (milk, soy, 14 weeks) vs. solid food-FPIES (12 weeks).

### Diagnosis and referral information in FPIES

The majority were diagnosed clinically, with only 6 (5%) requiring oral diagnostic food challenge. The average number of acute FPIES episodes prior to diagnosis was 2.7 and 34 patients (31%) had at least one emergency room visit for FPIES symptoms prior to diagnosis. For these patients, 47% required IV hydration, 33% underwent laboratory evaluation, 26% received antiemetics, 26% received imaging (x-rays and/or ultrasounds), 14% were admitted to the hospital, 11% underwent specialty evaluation (gastroenterology or infectious disease), and 1 patient underwent a complete sepsis evaluation (blood culture and lumbar puncture).

**Figure 1:**
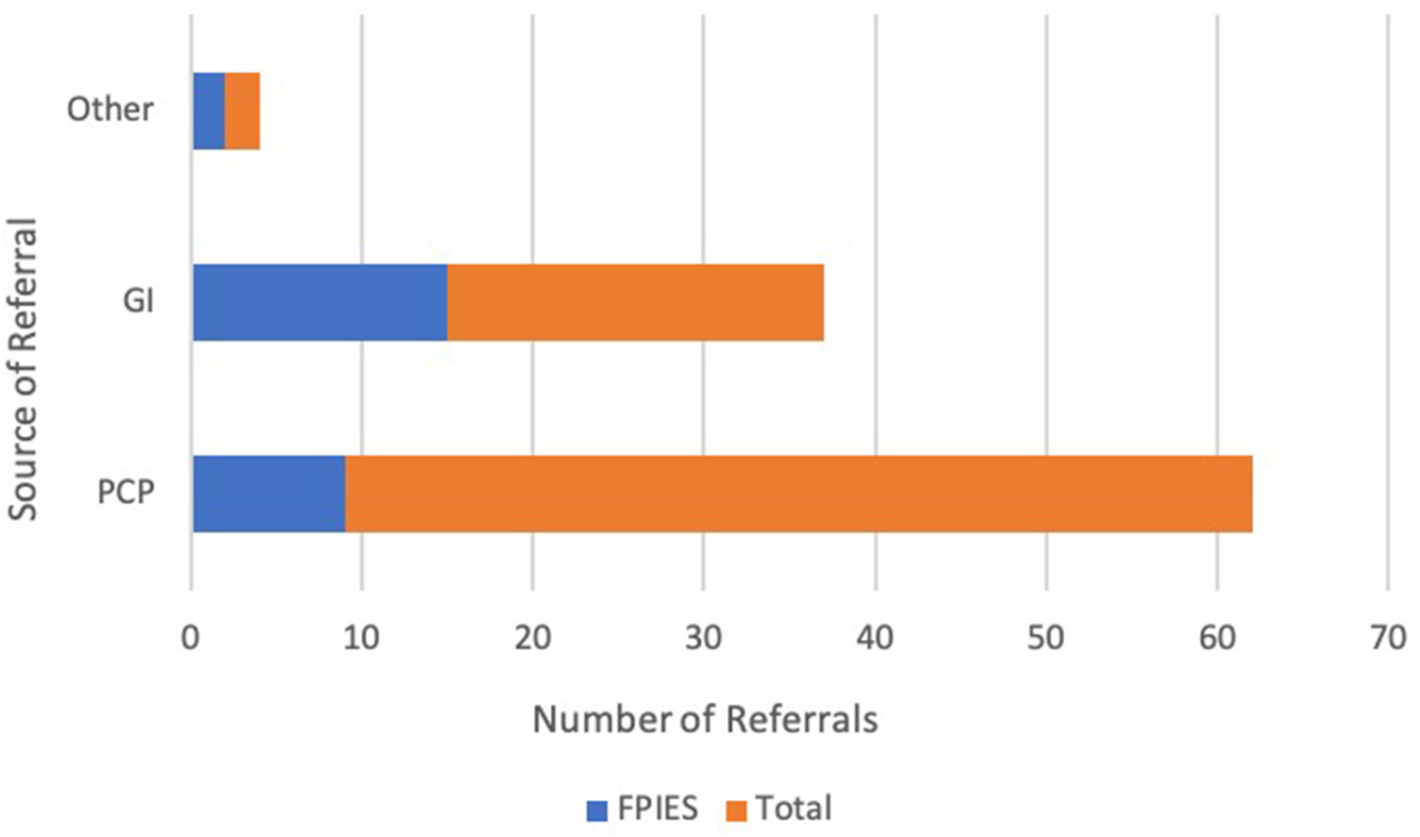
Number of total referrals for evaluation in whom FPIES was diagnosed and number of those referrals specifically made for concern of FPIES from each specialty.

For patients with referral information available (*n* = 78), 68% were from the primary pediatrician and 28% from pediatric gastroenterology. The most common reason for referral was for evaluation of IgE-mediated food allergy (53%), followed by FPIES (33%). Other reasons for referral included feeding difficulties, atopic dermatitis, urticaria, immunodeficiency, and medication allergy. Of the patients referred specifically for FPIES, 60% were from pediatric gastroenterology and 37% were from the primary pediatrician. A breakdown of number of total referrals and FPIES referrals is shown in Figure 1.

### Food triggers and other atopic diseases in FPIES

**Figure 2:**
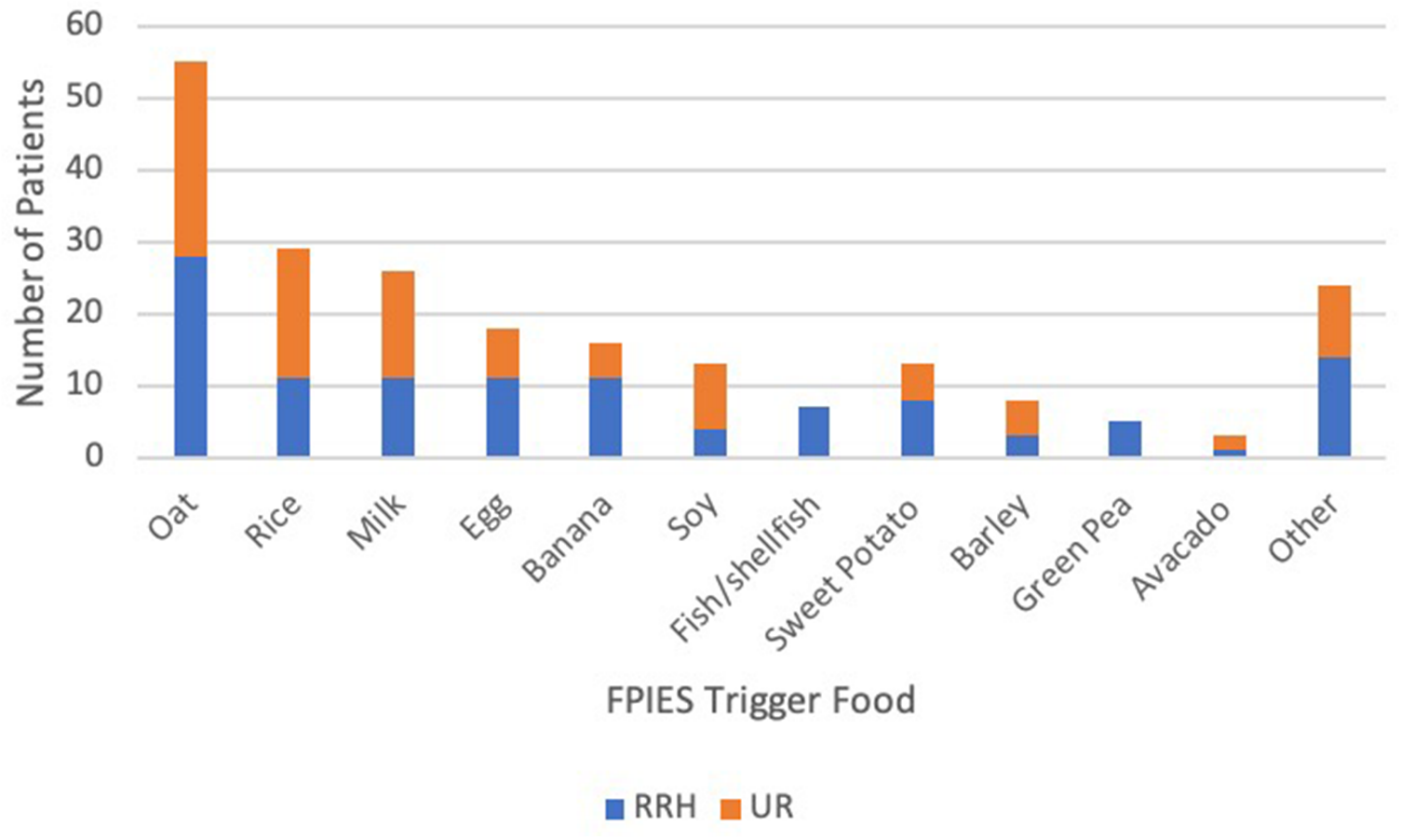
**Identified FPIES trigger food and number of patients at each cohort.** Other foods that were seen 2 or less patients included corn, meat (chicken, pork, beef, lamb), white potato, coconut, pear, apple, peanut, blueberry, chickpea, mango, cashew, carrot, cauliflower, sunflower, and watermelon.

**Figure 3:**
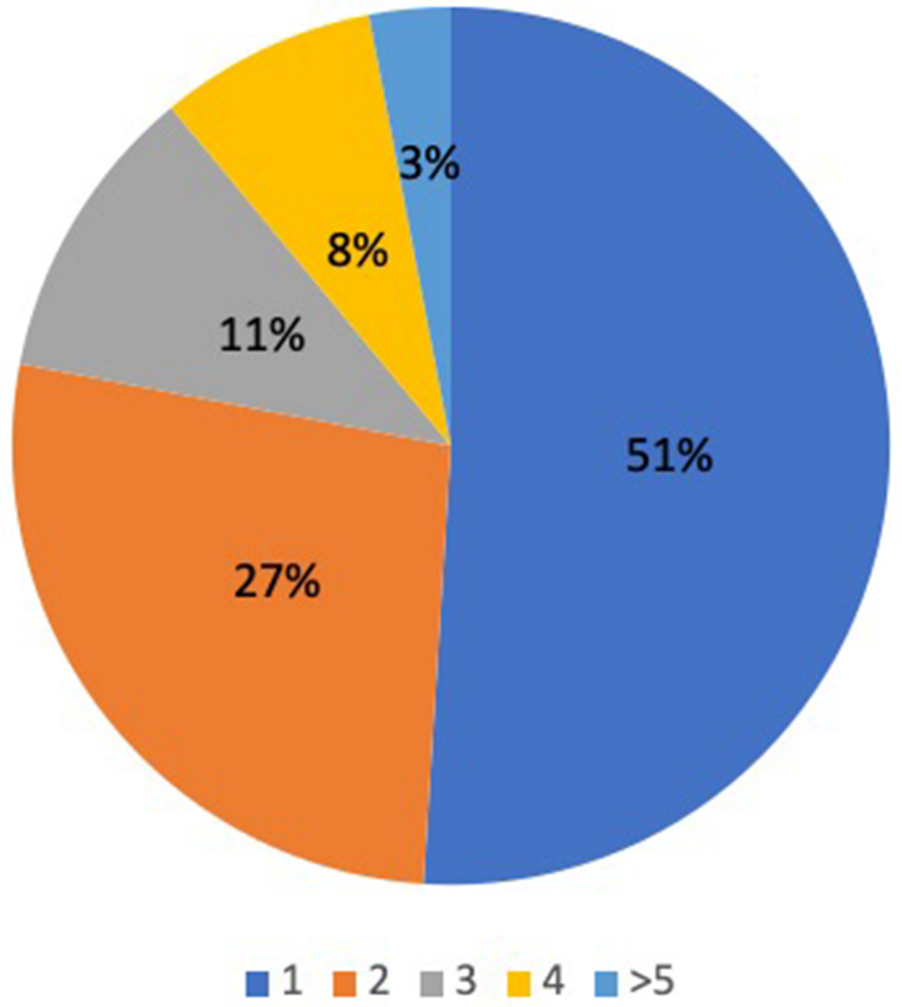
Total number of FPIES trigger foods among the cohort..

A total of 30 different trigger foods were identified and are shown in Figure 2. The most common triggers were oat (50%), rice (26%), cow's milk (24%), egg (16%). Most patients reacted to only one (51%) or two (27%) foods (Figure 3). A total of 102 patients (93%) underwent percutaneous skin prick testing with 9 (9%) showing sensitivity to their FPIES trigger food and 27 (25%) showing IgE-mediated sensitivity to any food. The most common skin prick positive food was egg (55%) and peanut (22%). Other foods to test positive on skin testing include milk, soy, wheat, tree nuts, oat, and rice. Egg was the most common FPIES trigger food to also have positive skin testing.

**Figure 4:**
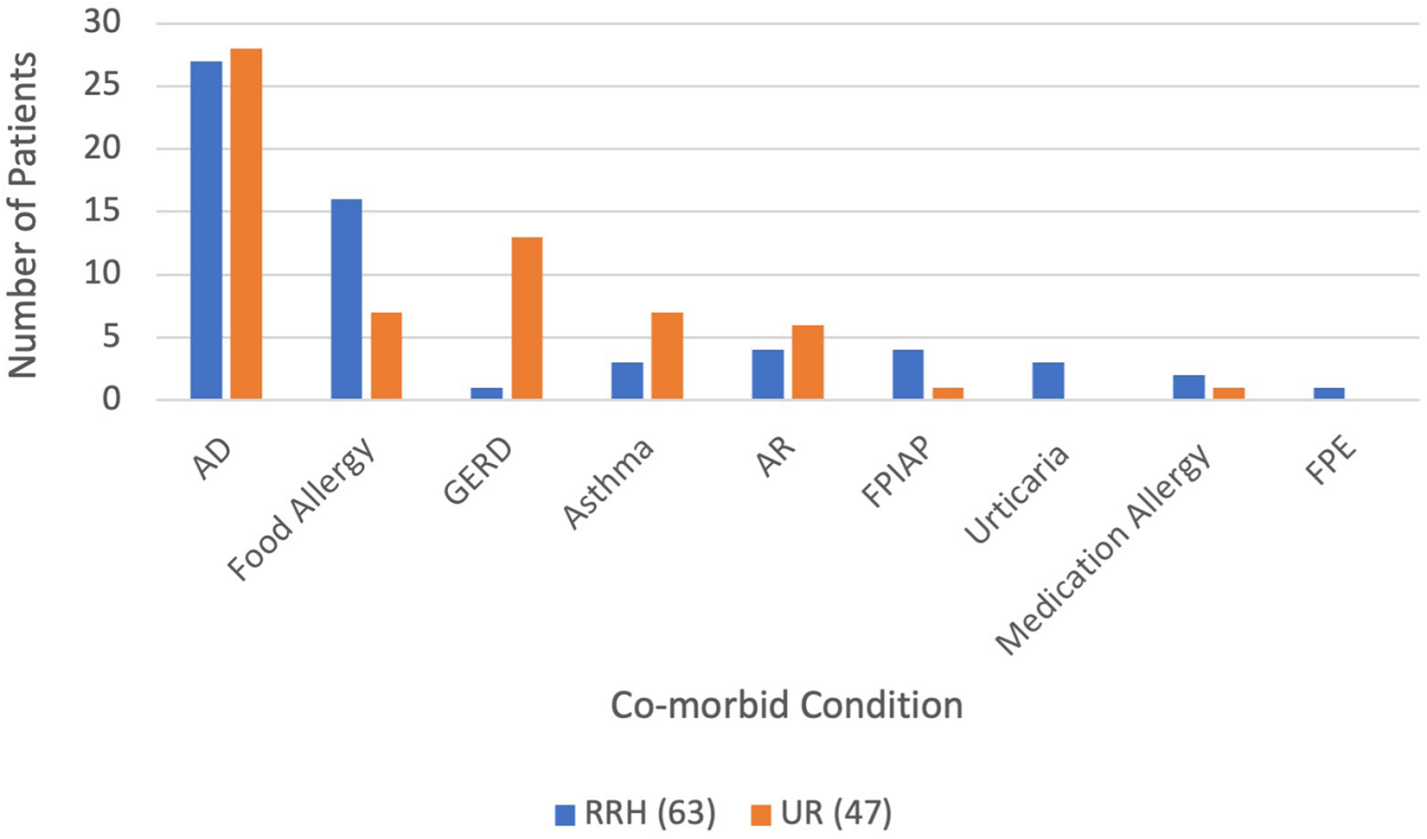
Atopic and gastrointestinal co-morbid conditions among cohorts. AD: atopic dermatitis, Food allergy: IgE-mediated food allergy, GERD: gastrointestinal reflux disease, AR: allergic rhinitis, FPIAP: food protein-induced allergic proctocolitis, FPE: food protein-induced enteropathy.

A family history (first degree relatives) of atopic disorders was prevalent (72%) with the most common being allergic rhinitis (46%), food allergy (25%), asthma (25%), and atopic dermatitis (17%), as shown in Table 2. A personal history of atopic disorders was also common; 50% with atopic dermatitis, 21% with IgE-mediated food allergy, 1% with asthma and allergic rhinitis. A complete breakdown of co-morbid conditions among the sites is shown in Figure 4. It appeared that gastroesophageal reflux disease (GERD) was common among FPIES patients seen at the UR.

**Table 2:** **Atopic family history in the FPIES cohorts**. Family history is defined as first degree relatives.

** **	UR (47)	RRH (63)	Total (110)
Atopic Family Hx	31 (66%)	48 (76%)	79 (72%)
Asthma	12 (26%)	15 (24%)	27 (25%)
Atopic Dermatitis	9 (19%)	10 (16%)	19 (17%)
Allergic Rhinitis	19 (40%)	32 (51%)	51 (46%)
Food Allergy	14 (30%)	13 (21%)	27 (25%)
Eosinophilic Esophagitis	2 (4%)	0	2 (2%)
Food Intolerance	3 (6%)	2 (3%)	5 (4.5%)
Venom Allergy	3 (6%)	1 (2%)	4 (4%)
Immunodeficiency	1 (2%)	0	1 (1%)
Drug Allergy	0	3 (5%)	3 (3%)
FPIES	1 (2%)	0	1 (1%)

FPIES, food protein-induced enterocolitis syndrome.

### Resolution of symptoms in FPIES

Within the time frame of the study, 19 patients underwent food challenge to assess tolerance development. The settings for these challenges included in 8 in office (42%), 5 at home (26%), and 6 inpatient (32%). Of those challenged, 15 patients (79%) had a negative challenge on the first attempt and the mean age of resolution was 31 months (range, 13–61 months). The tolerated foods included oat, rice, milk, apple, coconut, egg, peanut, and barely. The mean age of those who had a positive OFC was 43 months (range, 25–67 months). There was no statistically significant difference in resolution of symptoms between solid and liquid FPIES.

## Discussion

This study includes a relatively large cohort of FPIES patients among two different medical centers. Our study demonstrated a lag in diagnosis of FPIES (median 3 months), and although it was shorter than that found in previous studies (7, 8), it was significantly greater than that observed for patients with IgE-mediated food allergy. To our knowledge this is the first time that this comparison has been made. Additionally, during the time to diagnosis, FPIES patients had multiple episodes and many were seen in the ED, where about a quarter of patients underwent possibly unnecessary evaluation, such as imaging or laboratory evaluation. This follows with previously evaluated trends and shows a continued lack of recognition (9). Unlike, IgE-mediated food allergy, FPIES reactions are delayed and lack perceived classical signs of allergic reactions, such as urticaria, making the diagnosis more easily missed. Additionally, many of the FPIES trigger foods are not are readily perceived as “allergenic foods,” further hindering the recognition.

To our knowledge we are the first to examine referral patterns for FPIES within the United States, showing that the majority of patients were referred to an allergist for evaluation IgE-mediated allergy. Most patients were referred from their pediatrician, followed by gastroenterology. Of the patients specifically referred to an allergist for FPIES, the majority were from gastroenterology, suggesting that FPIES may be more familiar among this specialty than general practitioners. None of the patients who presented to the ED were referred from there for evaluation to an allergist. This shows that there is likely a lack of knowledge regarding FPIES among the general practitioners and the ED providers, who are usually the first to see the patients. The increased awareness of FPIES among these communities may help to shorten length to diagnosis and limit unnecessary evaluation.

The presentation and trigger foods were similar to those reported in prior studies (5–9). Interestingly GERD was a common comorbid condition in the UR cohort, which follows with previously identified trends (10). For the majority of patients, the diagnosis of GERD was a clinical diagnosis and, thus, there may be reporting errors.

The majority of FPIES patients were Caucasian, which follows with previously identified trends (6). This may suggest a genetic predisposition; however, we feel as that this is more suggestive of healthcare disparity. Minority races/ethnicities are known to have less access to health care. We postulate minority patients and those from low socioeconomic status, may be less likely to seek medical care or guidance after an FPIES episode and are less likely to receive care by a specialist. Given this, along with the overall lack of recognition, may hinder these groups from seeking medical care for FPIES episodes, and ultimately being diagnosed. With increased awareness, along with a focus on these at risk population, we may see more patients in these groups diagnosed with FPIES.

The recognition of FPIES has improved over the last few years; however, there remains a lack of recognition, especially outside the allergy community. An anonymous survey of AAAAI members showed that 64% reported full understanding of FPIES, while a survey of Italian pediatricians showed only 12% had a full understanding, with 7% reporting they had never heard of FPIES (11, 12). Feuille et al. also surveyed general practitioners in the United States and found that 67% reported some understanding of FPIES (13). The survey also showed that many practitioners did not recognize the full spectrum of symptoms related to FPIES and less than 20% of participants could recognize acute and chronic FPIES cases (14).

These previous studies, along with our study demonstrating the lack of recognition of FPIES outside of the specialty community, reinforces the need for continued efforts to increase awareness among all medical providers. Education of FPIES among a variety of health care providers, especially emergency room physicians and pediatricians, is vital to improving early recognition as these are typically the first providers to see these patients. This can be achieved initially by incorporating FPIES, as well as other non-IgE mediated food allergies, into medical school and graduate medical education curriculum. Discussions on non-IgE-mediated food allergies should be continued on the national dialogue, including conferences and research. This can help to improve awareness.

Obtaining a correct and prompt FPIES diagnosis is necessary for a number of reasons. Firstly, FPIES episodes are associated with increased anxiety in both the child and caregiver, as well as decreased quality of life in the child (14). There is also the financial cost, including dietary modifications, such as hypoallergenic formula, and the health care burden including multiple health care visits, including with a variety of specialists as well as with ED which not uncommonly comprise unnecessary evaluations. Appropriate and timely diagnosis can help to alleviate unnecessary healthcare costs and improve mental health for families. An accurate diagnosis allows for families to establish care with an allergist and receive appropriate FPIES care. This may include discussion on avoidance foods, introduction of new foods, appropriate formula selection when appropriate, and evaluation by a dietician to ensure proper nutritional needs are being met. Improving recognition of FPIES is vital as delayed diagnosis may result inappropriate evaluations, food avoidance, and parental anxiety.

Strengths of this study include its relatively large cohort from multiple institutions. Additionally, this is the first study to our knowledge to compare FPIES patients with other food allergies. Limitations include the study being retrospective in nature and relying on the medical records, which were not fully complete for each patient.

Overall, this large cohort of FPIES patients demonstrates the continued lack of recognition, especially among general practitioners and ED providers. Education of those who typically see these patients initially (emergency medicine and primary physicians) is imperative to help increase prompt diagnosis and improve care of our patients.

## Data Availability

The raw data supporting the conclusions of this article will be made available by the authors, without undue reservation.
